# Supplementary material for: Daily caloric restriction limits tumor growth more effectively than caloric cycling regardless of dietary composition
Source: Nat Commun. 2021 Oct 27;12:6201. doi: 10.1038/s41467-021-26431-4 (PMC8551193; doi:10.1038/s41467-021-26431-4)
Supplement: Supplementary file 1 — Supplementary Information [file 41467_2021_26431_MOESM1_ESM.pdf]

Supplementary Information

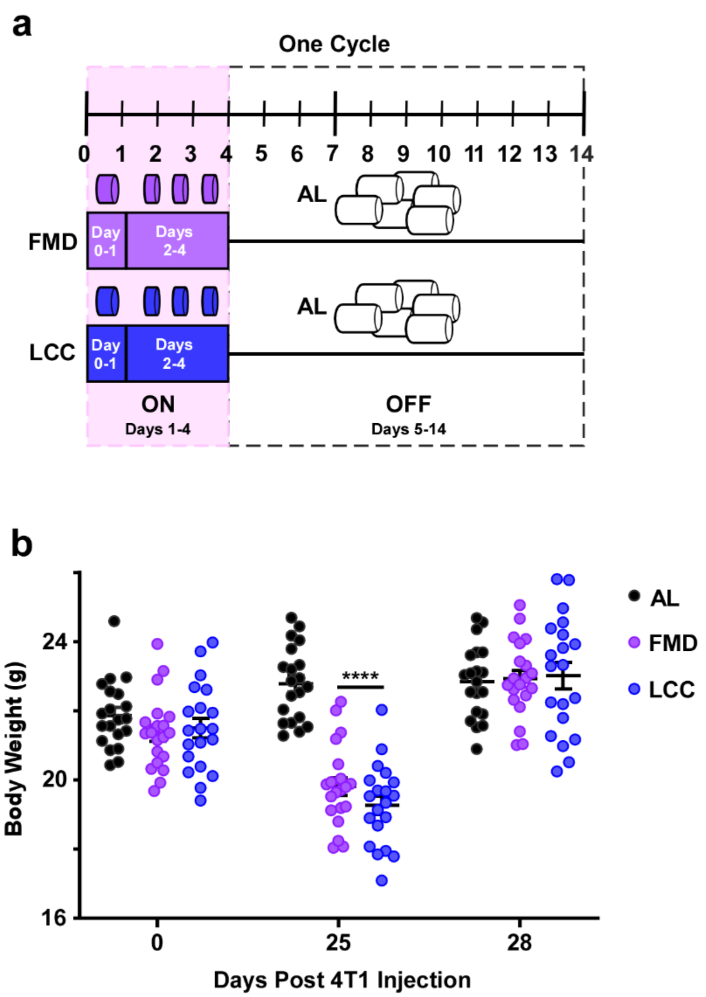

Supplementary Figure 1. Implementation of low caloric cycles (FMD & LCC).
(**a**) Experimental layout. This feeding regimen is comprised of two parts: a 4-day period of very low-calorie intake, followed by a 10-day refeeding period with ad libitum (AL) access to the standard laboratory chow (AIN-93G). Mice had a step-down reduction in daily food allotment during the 'ON' portion of the feeding regimen, whether on the fasting mimicking diet (FMD) or its isocaloric equivalent AIN-93G (LCC). On the first day (Day 0-1) of the cycle, mice received 50% of the amount of food (either FMD or LCC) given to *ad libitum* (AL)-fed mice, and for the remaining 3 days (days 2-4), they were further restricted by receiving 30% of the calories given to AL mice. Mice are then returned to AL feeding on AIN-93G diet, dubbed the 'OFF' portion, for the remainder of the 14-day cycle (Days 5-14). (**b**) Individual bodyweights following 4T1 injection: at the time of injection (day 0), day 25, and day 28. Data is represented as a scatter plot with mean values ± SEM. One-way ANOVA with Tukey post hoc analysis was used to determine statistical significance with **** $p < 0.0001$ compared to AL. **b** AL, n = 20; FMD, n = 19; LCC, n = 19 mice per treatment group. Source data are provided as a Source Data file.

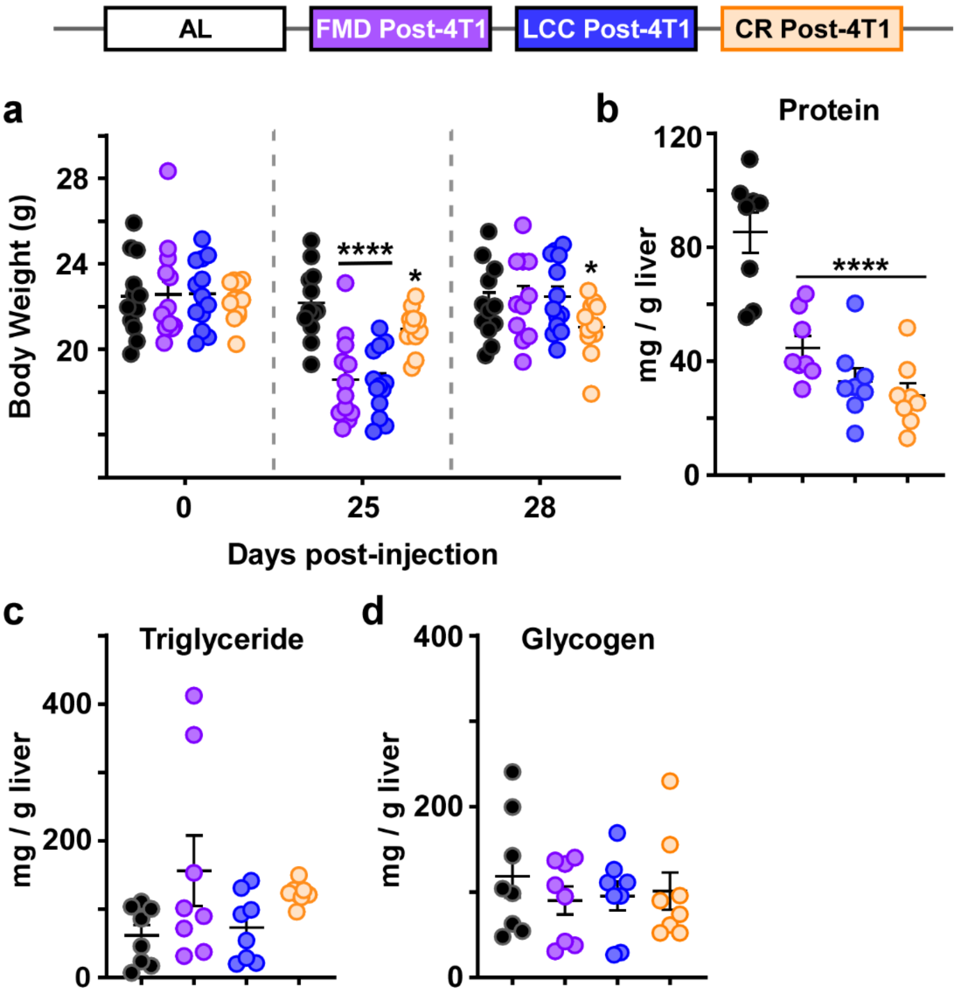

Supplementary Figure 2. Changes in bodyweight and liver content in mice undergoing caloric cycling or daily CR initiated after 4T1 implantation.

(**a**) Individual bodyweights following 4T1 injection: at the time of injection (day 0), day 25, and day 28. (**b**) Liver protein content. (**c**) Liver triglyceride content. (**d**) Liver glycogen content. Data is represented as a scatter plot with mean values ± SEM. One-way ANOVA with Tukey post hoc analysis was used to determine statistical significance with * $p < 0.05$, **** $p < 0.0001$ compared to AL. **a** AL, n = 13; FMD, n = 12; LCC, n = 13; CR, n = 13. **b-d** AL, n = 8; FMD, n = 8; LCC, n = 8; CR, n = 8 mice per treatment group. Source data are provided as a Source Data file.

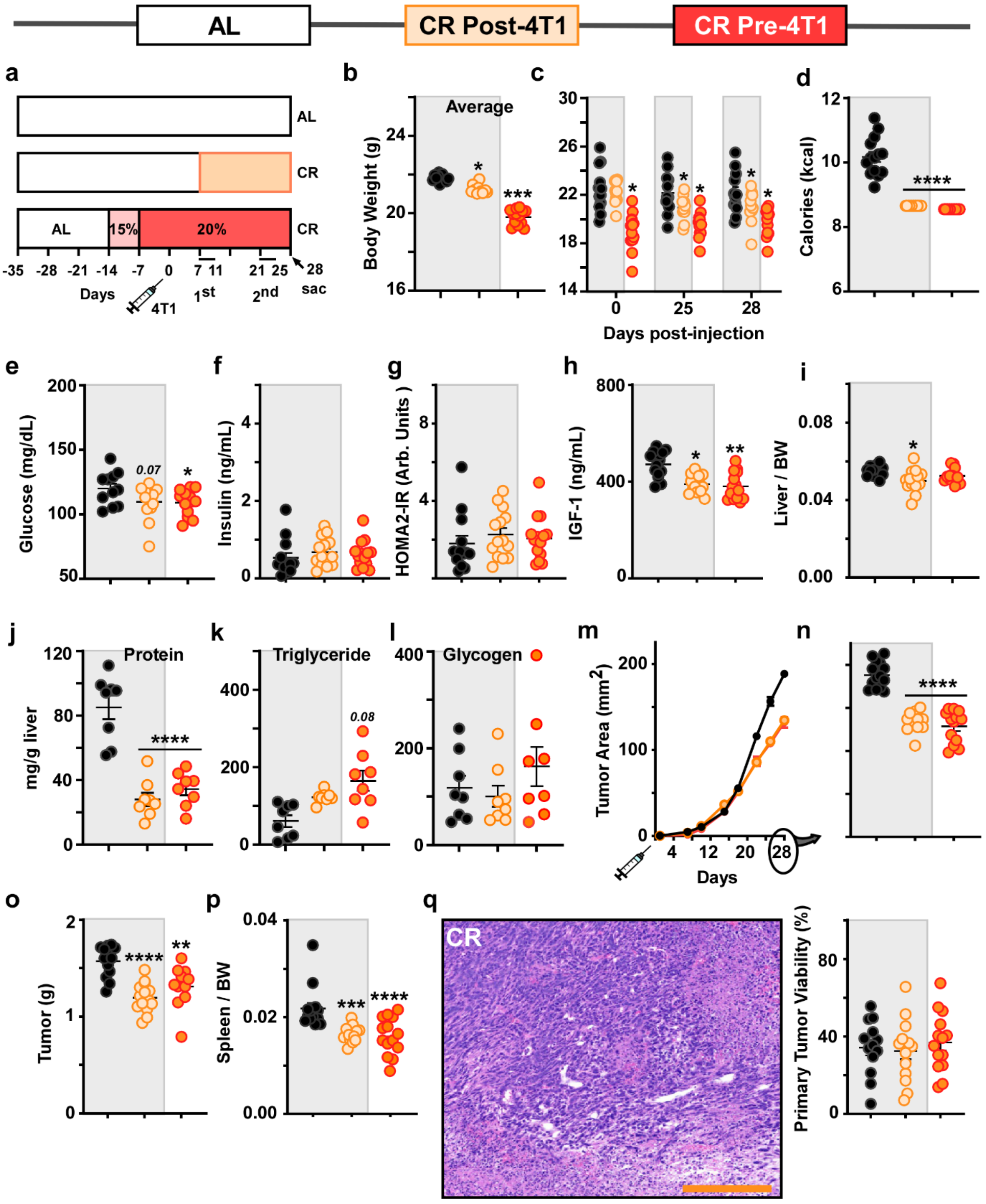

Supplementary Figure 3. Daily CR delays primary tumor growth equally, whether initiated pre- or post-4T1 injection.

(**a**) Experimental Layout. AL-fed BALB/cJ females underwent a step-down reduction in calories to 20% CR. Two weeks later, mice were injected with 4T1 cells (10^6 cells/mL) at day 0 and remained on daily CR until tissue collection at day 28. It is important to clarify, the daily CR pre-4T1 feeding regimen was conducted as an additional study arm concurrently with the treatment groups presented in Figure 2. We have included some of the findings from Figure 2 and the CR pre-4T1 group in order to better compare across all treatment groups. As such, the AL and CR post-4T1 groups presented in Figure 2 are the same as those shown in Supplementary Figure 3 (gray box). The treatment group of CR pre-4T1 was separated for manuscript clarity. (**b**) Average body weight and (**c**) individual body weights following 4T1 injection: at the time of injection (day 0), day 25, and day 28. (**d**) Average calories consumed over the course of the study (days 7-28). (**e**) Glucose levels, (**f**) serum insulin levels, (**g**) HOMA2-IR, and (**h**) serum IGF-1 levels collected at day 28. (**i**) Liver weight per unit of body weight. (**j**) Liver protein content, (**k**) liver triglyceride content, and (**l**) liver glycogen content. (**m**) Growth rate of primary tumor and (**n**) individual tumor area at day 28. (**o**) Tumor weight and (**p**) spleen weight per unit of body weight. (**q**) Representative image of a primary tumor developed under CR, including both viable and necrotic areas [H&E staining, original magnification 200X] and quantification of primary tumor viability (right panel). Scale bar = 100 μm. Most of the data are represented as scatter plots with mean values ± SEM. One-way ANOVA with Tukey post hoc analysis was used to determine statistical significance with * $p < 0.05$, ** $p < 0.01$, *** $p < 0.001$, **** $p < 0.0001$ compared to AL. **b-d, h, i**, **m-q** AL, n = 13; CR post-4T1, n = 13; CR pre-4T1, n = 13. **e-g** AL, n = 12; CR post-4T1, n = 13; CR pre-4T1, n = 13. **j-l** AL, n = 8; CR post-4T1, n = 8; CR pre-4T1, n = 8 mice per treatment group. Source data are provided as a Source Data file. BW, body weight; Arb units, arbitrary units.

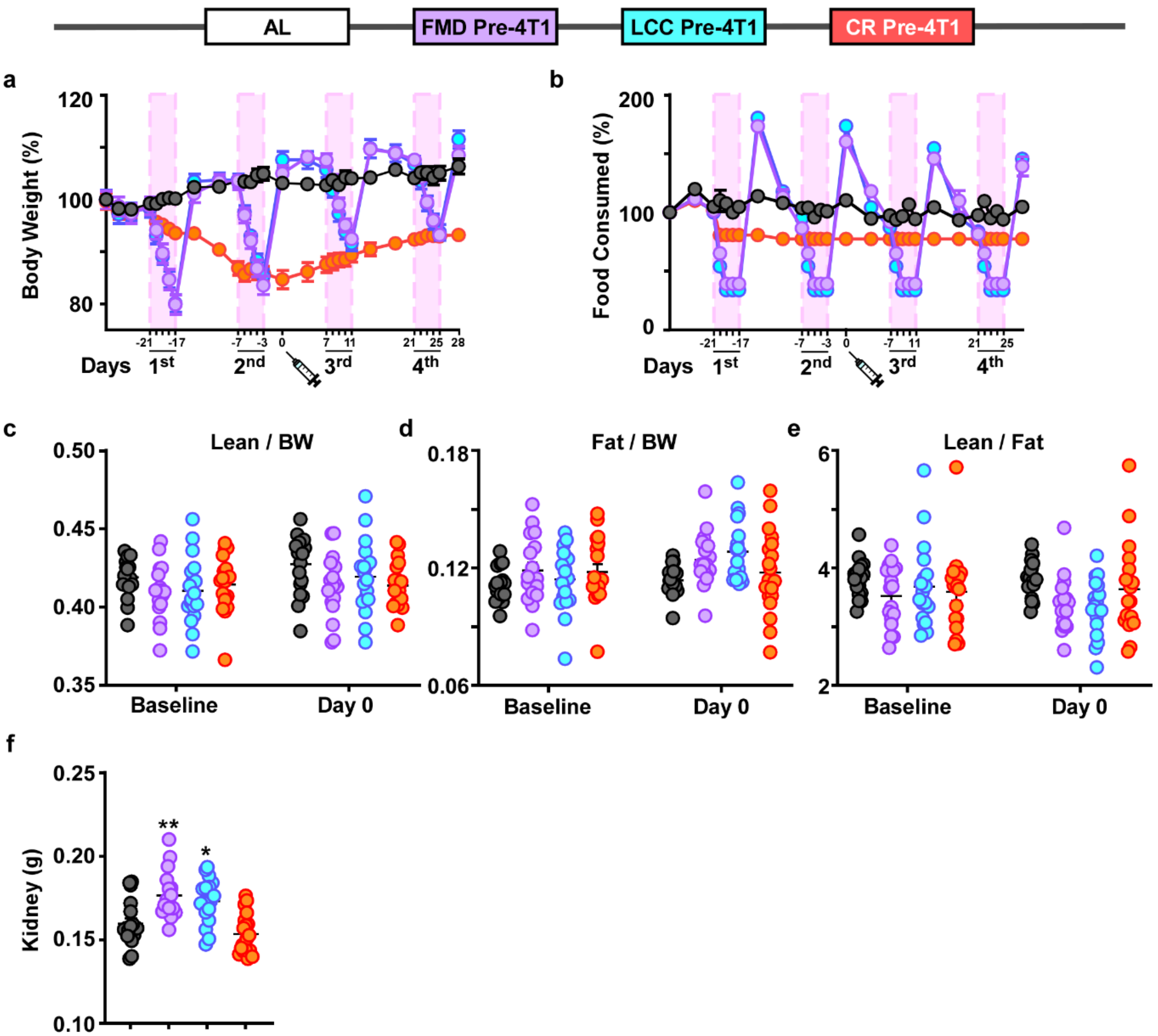

Supplementary Figure 4. Changes in physiological parameters in response to daily CR or caloric cycling initiated prior to 4T1 implantation.

(**a**) Percent change in bodyweight and (**b**) food consumption from baseline during the course of the study. Lean and fat mass were determined via NMR, conducted at baseline and at tumor implantation (day 0). (**c**) Lean and (**d**) fat mass per unit of body weight (BW). (**e**) The ratio of lean-to-fat mass. (**f**) Kidney mass at day 28. Most of the data are represented as scatter plots with mean values ± SEM. One-way ANOVA with Tukey post hoc analysis was used to determine statistical significance with * $p < 0.05$, ** $p < 0.01$, *** $p < 0.001$compared to AL. **a-f** AL, n = 18; FMD pre-4T1, n = 17; LCC pre-4T1, n = 18; CR pre-4T1, n = 19 mice per treatment group. Source data are provided as a Source Data file

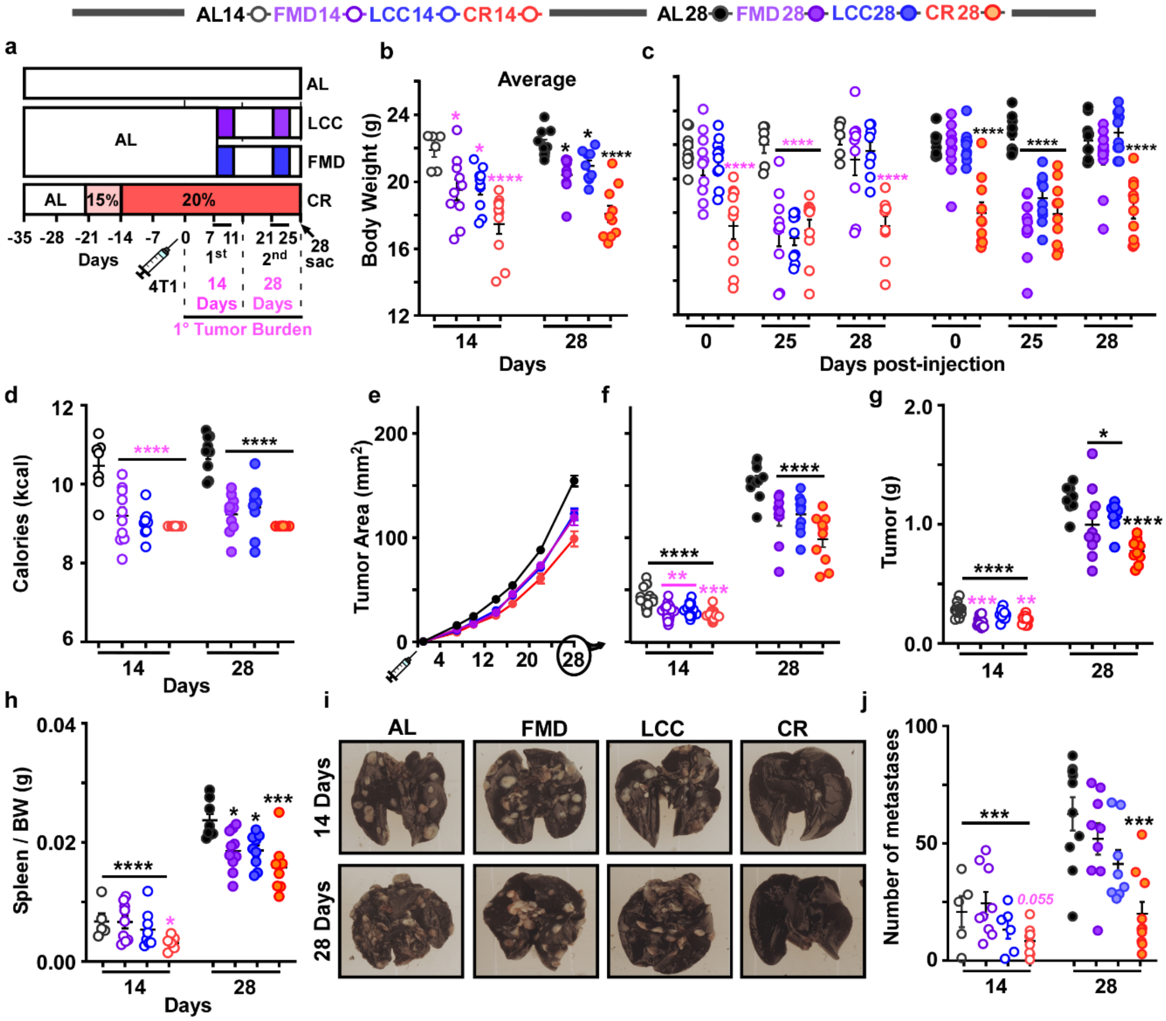

Supplementary Figure 5. Continuation of daily CR following removal of primary tumor leads to the greatest reduction in metastatic burden.

(**a**) Experimental layout. Mice were kept on AL for 14 days (days -35 to -21), at which point a subset of animals was given CR in a step-down fashion. At day 0, mice on 20% CR and AL-fed mice were injected with 4T1 breast cancer cells (10^6 cells/mL) and at day 7, mice on AL were randomized into three groups: They were either maintained on AL or subjected to the FMD or LCC regimen for the first 'ON' cycle (day 7-11). At day 14, half the mice in each dietary group (AL, FMD, LCC, 20% CR) had the primary tumor removed and resumed their dietary regimen. (**b**) Average body weight and (**c**) individual bodyweights following 4T1 injection: at the time of injection (day 0), day 25, and day 28. (**d**) Average caloric intake in mice with tumor burden for 14 days (open circles) or 28 days (closed circles). (**e**) Rate of tumor growth. (**f**) Tumor area at day 14 and day 28. (**g**) Tumor mass at days 14 and 28. (**h**) Spleen mass per unit of body weight at day 28 in mice with tumor burden for 14 days or 28 days. (**i**) Representative images of india ink-stained lungs of mice with 14- and 28-day tumor burden. (**j**) Macroscopic quantification of lung metastases after 14- and 28-day tumor burden [india ink-stained lungs]. Most of the data

are represented as scatter plots with mean values ± SEM * $p < 0.05$, ** $p < 0.01$, *** $p < 0.001$, **** $p < 0.0001$ compared to AL-fed mice with tumor burden for either 28 days (Black) or 14 days (Pink). Differences between groups were calculated by two-way ANOVA. **a-c** AL-14 days, n = 7; FMD-14 days, n = 10; LCC-14 days, n = 10; CR-14 days, n = 10; AL-28 days, n = 10, FMD-28 days, n = 10; LCC-28 days, n = 10; CR-28 days, n = 10. **d,f,g** n = 10 mice per treatment group. **h** AL-14 days, n = 5; FMD-14 days, n = 9; LCC-14 days, n = 8; CR-14 days, n = 6; AL-28 days, n = 8, FMD-28 days, n = 10; LCC-28 days, n = 9; CR-28 days, n = 8. **j** AL-14 days, n = 5; FMD-14 days, n = 9; LCC-14 days, n = 6; CR-14 days, n = 8; AL-28 days, n = 10, FMD-28 days, n = 9; LCC-28 days, n = 8; CR-28 days, n = 10. Source data are provided as a Source Data file

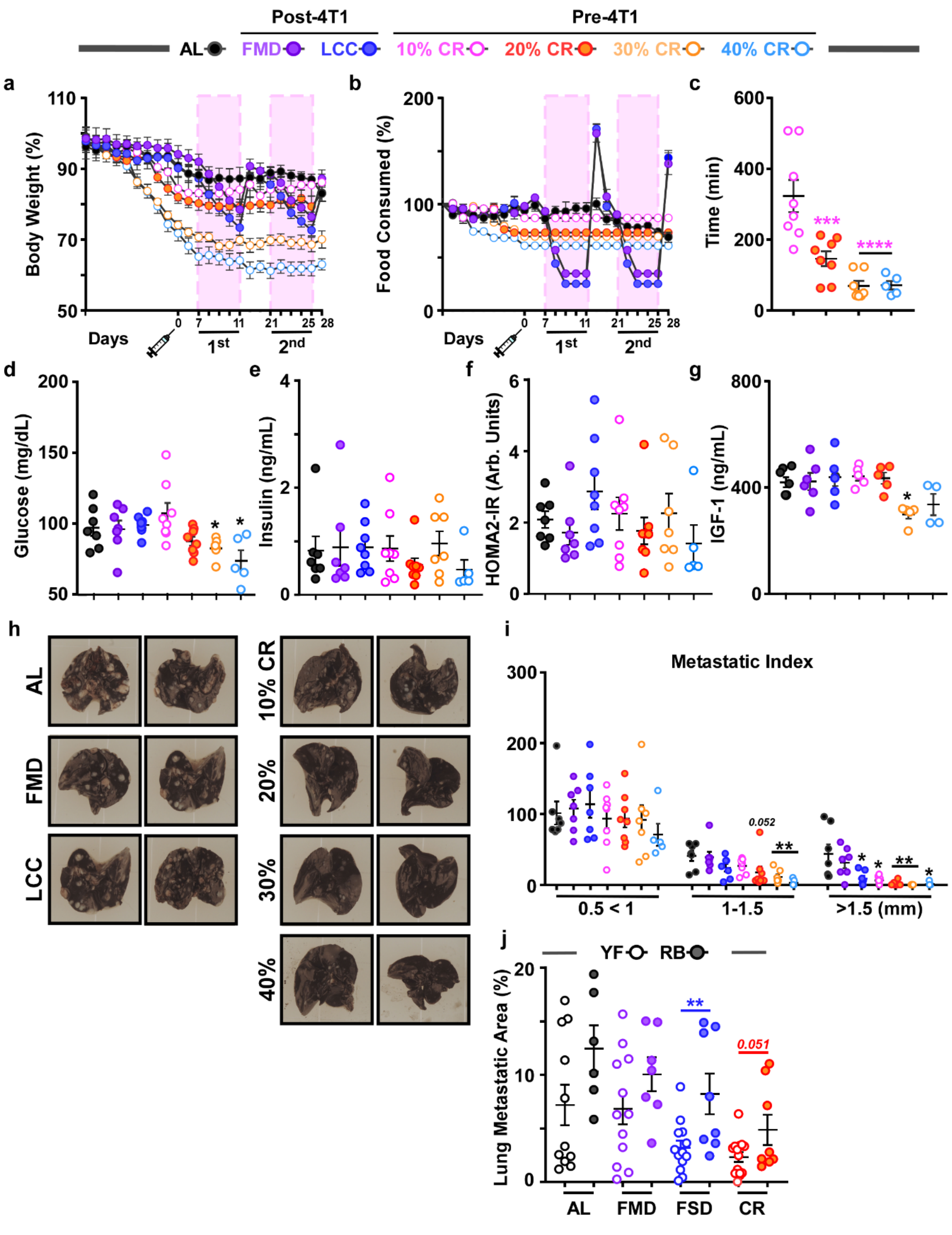

Supplementary Figure 6. Increasing stringency of daily CR leads to lower metastatic burden in post-reproductive females.

(**a**) Percent change in bodyweight and (**b**) food consumption from baseline during the course of the study. (**c**) Time to consume food at 10-40% CR, with significance compared to 10% CR was performed 25 days after tumor injection. (**d**) Glucose levels, (**e**) serum insulin levels, (**f**) HOMA2-

IR, and (**g**) serum IGF-1 levels collected at day 28. (**h**) Representative images demonstrating the appearance of white masses in india ink-stained lungs, indicative of metastases. (**i**) Lung metastases were scored in a blinded fashion and divided into three groups based on size: 0.5<1 mm, 1<1.15 mm, and >1.5 mm. Larger tumors have a more advanced histological grade and greater metastatic index. (**j**) Effect of feeding regimens (AL, FMD, LCC, 20% CR) on the percentage of metastatic area within the lung in young females (YF, open circles) versus retired breeders (RB, filled circles). Bars represent mean values ± SEM. One-way ANOVA with Tukey post hoc analysis was used to determine statistical significance with * $p < 0.05$, ** $p < 0.01$, *** $p < 0.001$, **** $p < 0.0001$ compared to AL (Black), 10% CR (Pink), or young FSD (Blue). **a,b** AL, n = 7, FMD, n = 7; LCC, n = 8; 10% CR, n = 6; 20% CR, n = 7; 30% CR, n = 7; 40% CR, n = 6. **c** 10% CR, n = 8; 20% CR, n = 8; 30% CR, n = 7; 40% CR, n = 5. **d-f** AL, n = 7, FMD, n = 7; LCC, n = 8; 10% CR, n = 8; 20% CR, n = 8; 30% CR, n = 7; 40% CR, n = 5. **g** AL, n = 7, FMD, n = 6; LCC, n = 6; 10% CR, n = 5; 20% CR, n = 5; 30% CR, n = 5; 40% CR, n = 4. **i** AL, n = 7, FMD, n = 7; LCC, n = 7; 10% CR, n = 8; 20% CR, n = 8; 30% CR, n = 7; 40% CR, n = 5. **j** AL-YF, n = 11; AL-RB, n = 6; FMD-YF, n = 12; FMD-RB, n = 7; LCC-YF, n = 12; LCC-RB, n = 8; CR-YF, n = 14; CR-RB, n = 8. Source data are provided as a Source Data file. Arb units, arbitrary units.

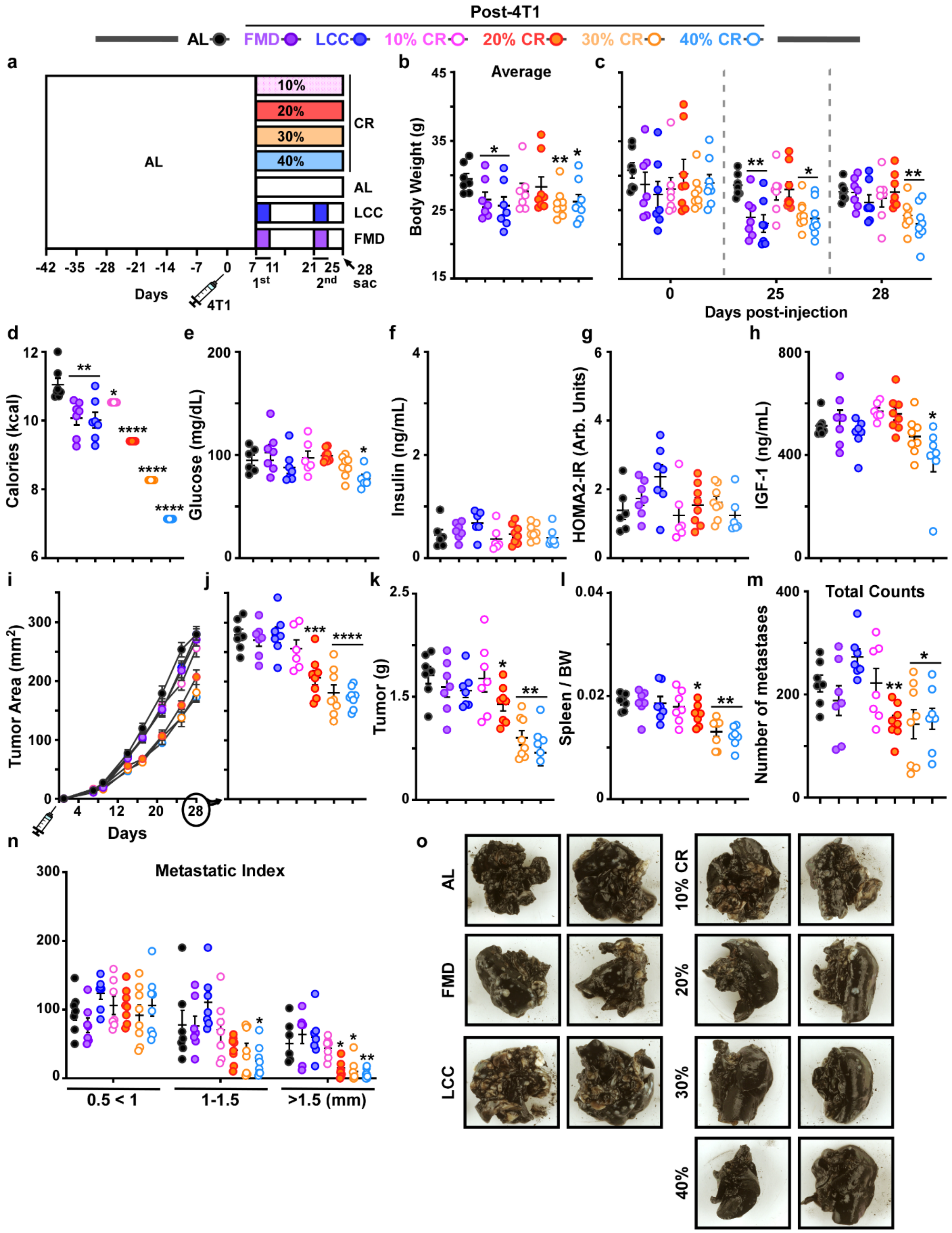

Supplementary Figure 7. Increasing stringency of CR post-4T1 implantation is effective at delaying primary tumor growth in middle-aged female mice.

(**a**) Experimental layout. 42-week-old AL-fed BALB/cJ females were injected with 4T1 cells (10^6 cells/mL) at day 0. Beginning at day 7, mice remained on AL or simultaneously were either switched to the specified doses of daily CR (10-40%) or subjected to two low-caloric cycles of FMD or LCC before tissue collection at day 28. (**b**) Average body weight and (**c**) individual bodyweights following 4T1 injection: at the time of injection (day 0), day 25, and day 28. (**d**) Average calories consumed over the course of the study (days 7-28). (**e**) Glucose levels, (**f**) serum insulin levels, (**g**) HOMA2-IR, and (**h**) serum IGF-1 levels collected at day 28. (**i**) Growth rate of primary tumor and (**j**) individual tumor area at day 28. (**k**) Tumor weight and (**l**) spleen weight per unit of body weight (BW). (**m**) Total number of lung metastases. (**n**) Lung metastases were scored in a blinded fashion and divided into three groups based on size: 0.5<1 mm, 1<1.15 mm, and >1.5 mm. Large tumors have a more advanced histological grade and greater metastatic index. (**o**) Representative images demonstrating the appearance of white masses in india ink-stained lungs, indicative of metastases. Most of the data are represented as scatter plots with mean values ± SEM. One-way ANOVA with Tukey post hoc analysis was used to determine statistical significance with * $p < 0.05$, ** $p < 0.01$, *** $p < 0.001$, **** $p < 0.0001$ compared to AL. **b-d** AL, n = 7; FMD, n = 7; LCC, n = 7; 10% CR, n = 7; 20% CR, n = 8; 30% CR, n = 8; 40% CR, n = 8. **e** AL, n = 6; FMD, n = 7; LCC, n = 7; 10% CR, n = 6; 20% CR, n = 8; 30% CR, n = 8; 40% CR, n = 6. **f** AL, n = 6; FMD, n = 7; LCC, n = 6; 10% CR, n = 6; 20% CR, n = 8; 30% CR, n = 8; 40% CR, n = 7. **g** as in (f) except 40% CR, n = 6. **h,k,l** as in (b-d) except 40% CR, n = 7. **i, j** AL, n = 7; FMD, n = 7; LCC, n = 7; 10% CR, n = 6; 20% CR, n = 8; 30% CR, n = 8; 40% CR, n = 8 mice per treatment group. **m, n** as in (b-d). Source data are provided as a Source Data file. Arb units, arbitrary units.

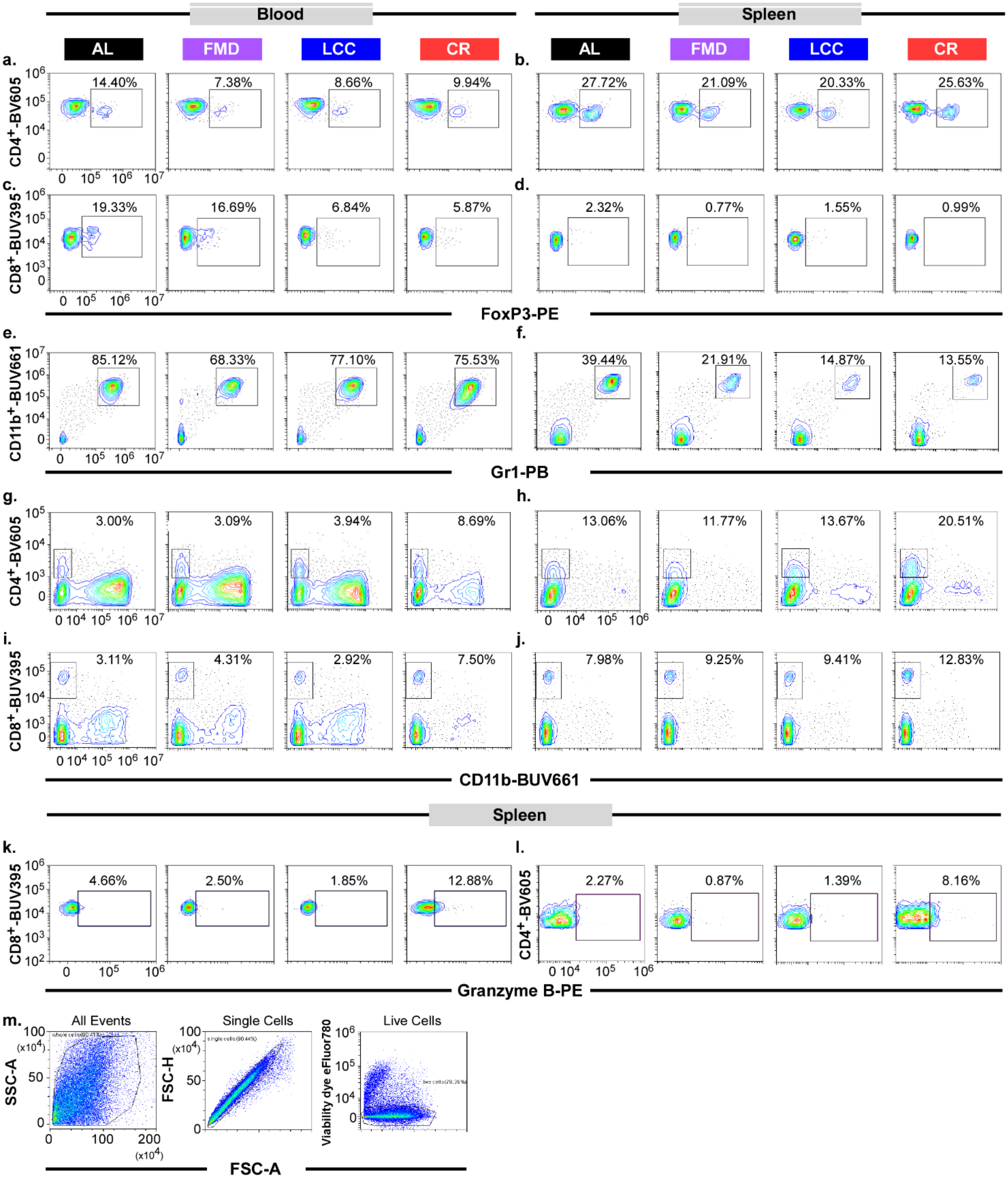

Supplementary Figure 8. Daily CR leads to a unique immune signature in response to 4T1.

FACS analysis for the data presented in Figure 6 of immune cells from blood and spleen. Detection of Tregs ($CD4^{+}$Foxp3 and $CD8^{+}$Foxp3) detected in the (**a,c**) blood and (**b,d**) spleen,

respectively. Detection of MDSCs in the (**e**) blood and (**f**) spleen. Detection of cytotoxic effector T cells ($CD4^+$ and $CD8^+$) from (**g,i**) blood and (**h,j**) spleen, respectively. (**k,l**) Detection of GrB^+ cytotoxic effector T cells ($CD4^+$ and $CD8^+$) from spleen. (**m**) Gating strategy; this strategy was applied for all the plots in the figure. Each graph is a representative plot from one mouse in each of the treatment groups, with n = 8-10 mice per treatment group.

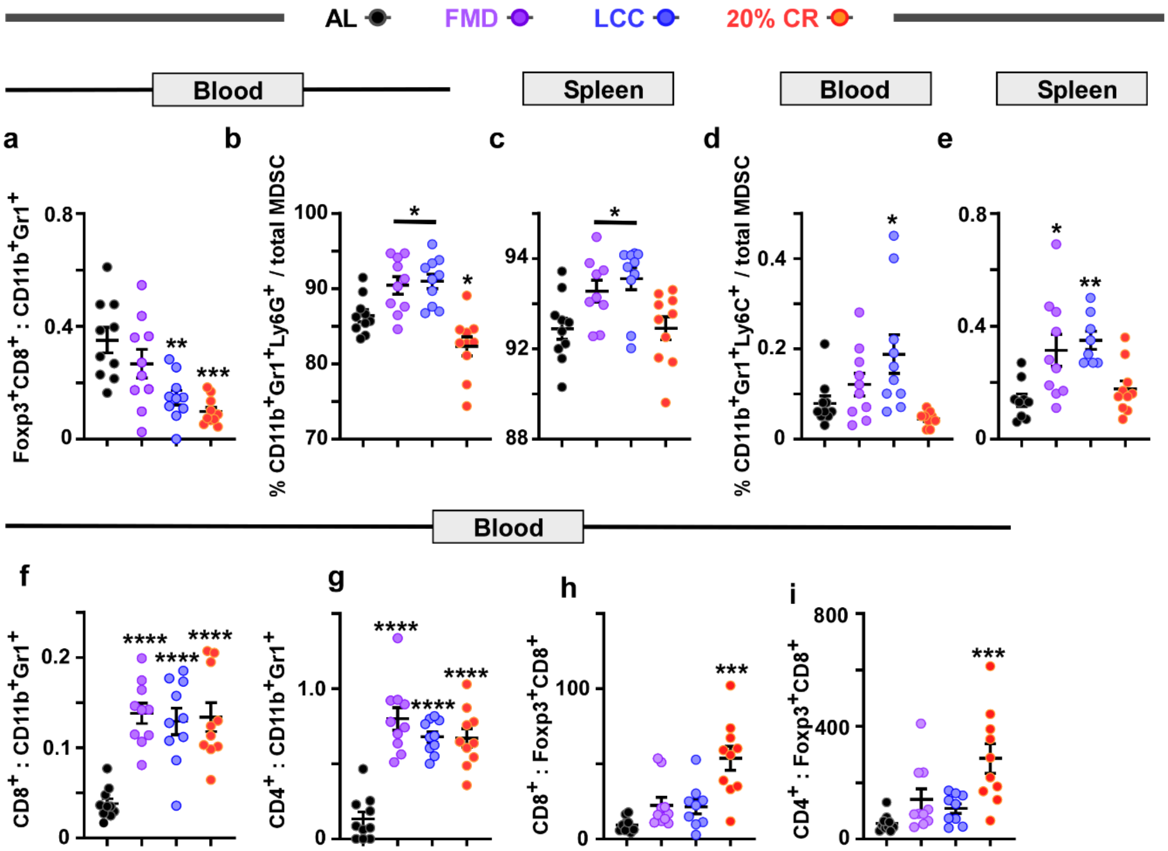

Supplementary Figure 9. Daily CR leads to a specific immune signature.

Flow cytometry analysis of various populations of immune cells identified in the peripheral blood and spleen. (**a**) Ratio of T regulatory $Foxp3^+CD8^+$ cells to $CD11b^+Gr1^+$ cells (MDSCs) in peripheral blood. (**b, c**) Percentage of $CD11b^+Gr1^+Lys6G^+$ (granulocytic-MDSCs) and (**d, e**) $CD11b^+Gr1^+Lys6C^+$ (monocytic-MDSCs) detected in the peripheral blood and spleen. (**f**) Ratio of effector $CD8^+$ to $CD11b^+Gr1^+$ cells (MDSCs). (**g**) Ratio of effector $CD4^+$ to $CD11b^+Gr1^+$ cells (MDSCs). (**h**) Ratio of effector $CD8^+$ to T regulatory $Foxp3^+CD8^+$ cells. (**i**) Ratio of effector $CD4^+$ to T regulatory $Foxp3^+CD8^+$ cells. Scatter plots represent mean values ± SEM. One-way ANOVA with Tukey post hoc analysis was used to determine statistical significance with * $p < 0.05$, ** $p < 0.01$, *** $p < 0.001$, **** $p < 0.0001$. **a-b, d, f, g** AL, n = 10; FMD, n = 10; LCC, n = 10; CR, n = 10. **c** AL, n = 10; FMD, n = 9; LCC, n = 10; CR, n = 10. **e** AL, n = 9; FMD, n = 10; LCC, n = 8; CR, n = 10. **h, i** AL, n = 10; FMD, n = 10; LCC, n = 9; CR, n = 10 mice per treatment group.

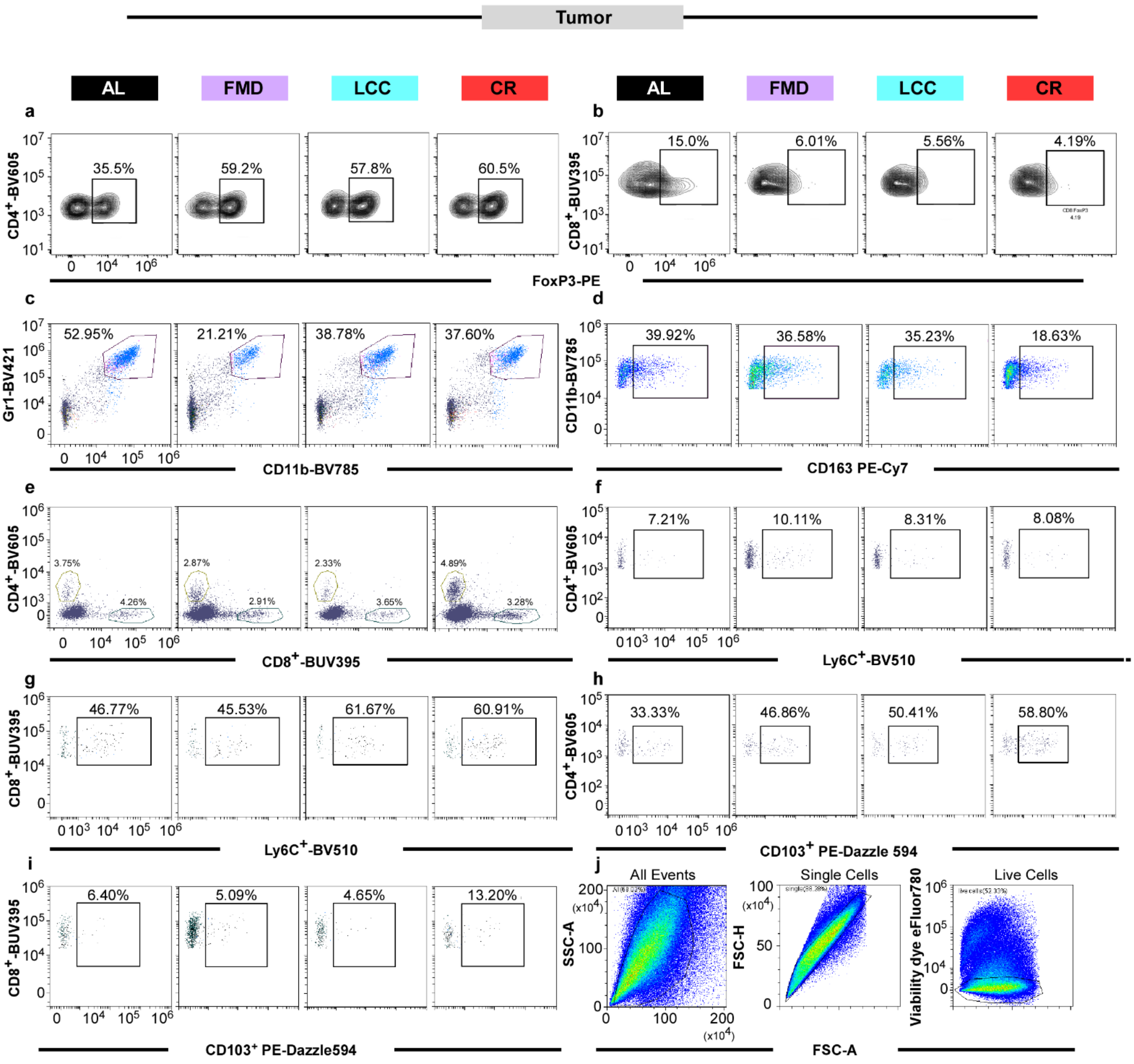

Supplementary Figure 10. FACS analysis of immune cells present in the primary tumor. (**a**,**b**) Detection of Tregs (CD4$^+$Foxp3 and CD8$^+$Foxp3). (**c**) Detection of MDSCs. (**d**) Detection of CD11b$^+$F480$^+$CD163$^+$ immunosuppressive cells. (**e**) Detection of cytotoxic effector T cells (CD4$^+$ and CD8$^+$). Detection of Lys6C$^+$ in (**f**) CD4$^+$ and (**g**) CD8$^+$ effector cells. Detection of CD103$^+$ cells in (**h**) CD4$^+$ and (**i**) CD8$^+$ effector cells. (**j**) Gating strategy; this strategy was applied for all the plots in the figure. Each graph is a representative plot from one mouse in each of the treatment groups, with n = 8-10 mice per treatment group.

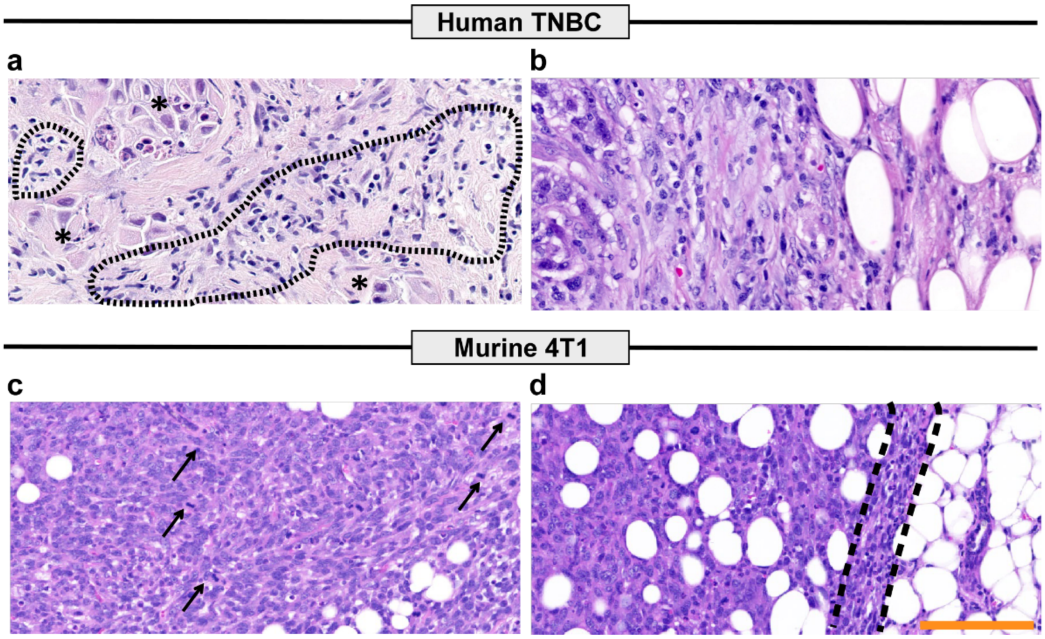

Supplementary Figure 11. Comparison between the histological appearance of a human TNBC and the murine 4T1 cell-derived tumors of the present study.

The images of illustrative breast cancer cases containing TILs were selected by 2 experienced pathologists from the International Immuno-Oncology Biomarker Working Group (chaired by R.S.) -www.tilsinbreastcancer.org- (R.S., P.G.E.). This group has demonstrated that the assessment of TILs is reproducible - doi: 10.1038/modpathol.2016.109.-. These representative images were selected to demonstrate that the evaluation of TILs using the Guidelines from the Working Group -doi: 10.1093/annonc/mdu450- cannot be extrapolated to the mice setting. (**a**) Human TNBC, featured by islets of carcinoma cells (asterisks) separated by fibrous stroma infiltrated by mononuclear host immune cells (predominantly lymphocytes or tumor infiltrating lymphocytes, TILs) (dotted curve shapes); (**b**) Invasive margin of a human TNBC. (**c**) Murine 4T1 cell-derived tumor showing several mitoses that do not display any intervening stroma. (**d**) This tumor directly infiltrates the contiguous mammary adipose tissue at the invasive margin, where the adipocytes are surrounded by clusters of mononuclear and polymorphonucleate immune cells (dashed lines), which are not considered to be TILs [**a-d**, H & E staining, original magnification 400X]; Scale bar = 100 μm.

Supplementary Table 1: Macronutrient Composition.

Macronutrient composition of the AIN-93G diet and the plant-based, Fasting Mimicking Diet (FMD).

	**AIN-93G (LCC)***		**FMD**	
Total energy density	3.76 kcal/g		2.92 kcal/g	
	g/kg diet	% kcal	g/kg diet	% kcal
Carbohydrate	601.0	63.9	320.3	44.5
Protein	177.0	18.8	62.0	8.6
Total fat<br>(saturated)	72.0**<br>(11.5)	17.2<br>(2.75)	150.1<br>(28.2)	46.9<br>(8.8)
Hydrogel	--		330.9	
Fiber	50.0	--	76.2	--
Vitamin and mineral mix (NR-1)			5.0	
Vitamin mix (#94047)	10.0			
Mineral mix ($94046)	35.0			

* The AIN-93G diet contains 200 g of casein and 70 g of soybean oil/kg diet. Soybean oil has 16% saturated fat, 23% MUFA, and 58% PUFA. The AIN-93G diet also contains 2.5 g of choline bitartrate and 14 mg of TBHQ antioxidant/kg diet.
