# Supplementary material for: Daily caloric restriction limits tumor growth more effectively than caloric cycling regardless of dietary composition
Source: Nat Commun. 2021 Oct 27;12:6201. doi: 10.1038/s41467-021-26431-4 (PMC8551193; doi:10.1038/s41467-021-26431-4)
Supplement: Supplementary file 3 — Reporting summary [file 41467_2021_26431_MOESM3_ESM.pdf]

nature research

Corresponding author(s): Rafael de Cabo

Last updated by author(s): 2021/09/18

Reporting Summary

Nature Research wishes to improve the reproducibility of the work that we publish. This form provides structure for consistency and transparency in reporting. For further information on Nature Research policies, see our Editorial Policies and the Editorial Policy Checklist.

Statistics

For all statistical analyses, confirm that the following items are present in the figure legend, table legend, main text, or Methods section.

n/a	Confirmed	
☐	☒	The exact sample size (n) for each experimental group/condition, given as a discrete number and unit of measurement
☐	☒	A statement on whether measurements were taken from distinct samples or whether the same sample was measured repeatedly
☐	☒	The statistical test(s) used AND whether they are one- or two-sided<br>*Only common tests should be described solely by name; describe more complex techniques in the Methods section.*
☐	☒	A description of all covariates tested
☐	☒	A description of any assumptions or corrections, such as tests of normality and adjustment for multiple comparisons
☐	☒	A full description of the statistical parameters including central tendency (e.g. means) or other basic estimates (e.g. regression coefficient) AND variation (e.g. standard deviation) or associated estimates of uncertainty (e.g. confidence intervals)
☐	☒	For null hypothesis testing, the test statistic (e.g. F, t, r) with confidence intervals, effect sizes, degrees of freedom and P value noted<br>*Give P values as exact values whenever suitable.*
☒	☐	For Bayesian analysis, information on the choice of priors and Markov chain Monte Carlo settings
☒	☐	For hierarchical and complex designs, identification of the appropriate level for tests and full reporting of outcomes
☒	☐	Estimates of effect sizes (e.g. Cohen's d, Pearson's r), indicating how they were calculated

Our web collection on statistics for biologists contains articles on many of the points above.

Software and code

Policy information about availability of computer code

Data collection	Cytoflex platform for flow cytometry
Data analysis	GraphPad Prism v. 8.4.2., CytoExpert v. 2.3, FlowJo v. 10.8 , QuPath v. 0.2.2., and HOMA2 calculator v. 2.2.2. were used to analyze the data presented in the study.

For manuscripts utilizing custom algorithms or software that are central to the research but not yet described in published literature, software must be made available to editors and reviewers. We strongly encourage code deposition in a community repository (e.g. GitHub). See the Nature Research guidelines for submitting code & software for further information.

Data

Policy information about availability of data

All manuscripts must include a data availability statement. This statement should provide the following information, where applicable:

- Accession codes, unique identifiers, or web links for publicly available datasets
- A list of figures that have associated raw data
- A description of any restrictions on data availability

Resources, reagents and microscopy images are available from the corresponding author, Rafael de Cabo (decabora@mail.nih.gov), upon reasonable request. Data generated in this study are provided in the Supplementary Information/Source Data file.

Field-specific reporting

Please select the one below that is the best fit for your research. If you are not sure, read the appropriate sections before making your selection.

[x] Life sciences [ ] Behavioural & social sciences [ ] Ecological, evolutionary & environmental sciences

For a reference copy of the document with all sections, see nature.com/documents/nr-reporting-summary-flat.pdf

Life sciences study design

All studies must disclose on these points even when the disclosure is negative.

Sample size	No statistical methods were used to predetermine sample size; however, one of our recent publications has highlighted the importance of proper sample size estimation in order to ensure adequate power to detect significant group differences or treatment effects in physiological and metabolic outcomes while controlling the type I error rate (PMID: 34407413). Using these estimates, we employed a sample size sufficient to achieve adequate power.
Data exclusions	No data were excluded from the analysis.
Replication	All experiments were reproduced at least twice to reliably support conclusions stated in the manuscript.
Randomization	Mice were randomly divided into experimental groups and fed either a standard laboratory chow or subjected to a dietary intervention [calorie restriction, a plant-based diet (FMD), 4:10 feeding cycles]. No significant differences in body weight were observed at baseline.
Blinding	The investigators were blinded to group allocation during scoring of lung metastases, tumor sizing and mitotic activity, recording of body weight and food consumption, flow cytometry, biochemical markers, and organ weights.

Reporting for specific materials, systems and methods

We require information from authors about some types of materials, experimental systems and methods used in many studies. Here, indicate whether each material, system or method listed is relevant to your study. If you are not sure if a list item applies to your research, read the appropriate section before selecting a response.

Materials & experimental systems

n/a	Involved in the study	
[ ]	[x]	Antibodies
[ ]	[x]	Eukaryotic cell lines
[x]	[ ]	Palaeontology and archaeology
[ ]	[x]	Animals and other organisms
[x]	[ ]	Human research participants
[x]	[ ]	Clinical data
[x]	[ ]	Dual use research of concern

Methods

n/a	Involved in the study	
[x]	[ ]	ChIP-seq
[ ]	[x]	Flow cytometry
[x]	[ ]	MRI-based neuroimaging

Antibodies

Antibodies used	Serum insulin was measured using a mouse ultra-sensitive enzyme-linked immunosorbent assay (Catalog #90080; Crystal Chem, Downers Grove, IL). The source of antibodies used for flow cytometry were the following: CD4 BV605 (Biolegend, Catalog #100451); CD8a BUV395 (BD Biosciences Catalog #: 563786); FoxP3 PE (Invitrogen/eBioscience, Catalog #12-5773-82); CD11b BV785 (Biolegend, Catalog #101243) and CD11b BUV661 (BD Biosciences, Catalog #612977); Gr1 PB (Biolegend, Catalog # 108430) and Gr1 BV421 (BD Biosciences, Catalog # 562709); Ly6G PerCP Cy5.5 (BD Biosciences, Catalog #560602); Ly6C BV510 (Biolegend, Catalog #128033); CD103 PE/Dazzle c594 (Biolegend, Catalog #121430); CD163 PE Cy7 (Invitrogen/eBioscience Catalog #25-1631-82); F4/80 APC (Biolegend, Catalog #123116); Granzyme B PE (Invitrogen/eBioscience, Catalog #12-8898-82); Viability Dye eFluor780 (Invitrogen/eBioscience, Catalog #65-0865-18).
Validation	We relied on the validation statements on the manufacturer's website for each antibody.

Eukaryotic cell lines

Policy information about cell lines

Cell line source(s)	4T1 cells were purchased from American Type Culture Collection (ATCC) #CRL-2539.
Authentication	We did not independently authenticate this cell line.

Mycoplasma contamination	These cells tested negative for mycoplasma contamination.
Commonly misidentified lines (See ICLAC register)	No commonly misidentified cell lines were used in this study.

Animals and other organisms

Policy information about studies involving animals; ARRIVE guidelines recommended for reporting animal research

Laboratory animals	12- and 36-week-old BALB/6J female mice were procured from the Jackson Laboratory (Bar Harbor, ME).
Wild animals	No wild animals were used in the study.
Field-collected samples	No field collected samples were used in the study.
Ethics oversight	Animal protocols were approved by the Animal Care and Use Committee (277-TGB-2024) of the National Institute on Aging, National Institutes of Health.

Note that full information on the approval of the study protocol must also be provided in the manuscript.

Flow Cytometry

Plots

Confirm that:

- [x] The axis labels state the marker and fluorochrome used (e.g. CD4-FITC).
- [x] The axis scales are clearly visible. Include numbers along axes only for bottom left plot of group (a 'group' is an analysis of identical markers).
- [x] All plots are contour plots with outliers or pseudocolor plots.
- [x] A numerical value for number of cells or percentage (with statistics) is provided.

Methodology

Sample preparation	The sample preparation is described in the method section of the manuscript.
Instrument	Cytoflex platform, Beckman Coulter, Cytoflex-LX
Software	CytoExpert v. 2.3, FlowJo v. 10.8
Cell population abundance	These parameters are the topic of the current paper and are provided as data in the main-text figures and Supplemental figures.
Gating strategy	Supplemental Figures 8m and 11j provide the gating strategy. Moreover, the method section offers additional information with regard to the gating strategy.

- [x] Tick this box to confirm that a figure exemplifying the gating strategy is provided in the Supplementary Information.
